# Validation of the Arabic version of the Mental Health Literacy Scale (MHLS) in three Arab samples

**DOI:** 10.1371/journal.pone.0335126

**Published:** 2025-11-26

**Authors:** Fareeda Abo-Rass, Ora Nakash, Sarah Abu-Kaf, Bizu Gelaye, Hanan AboJabel, Anwar Khatib

**Affiliations:** 1 Takemi Program in International Health, Harvard T.H. Chan School of Public Health, Boston, Massachusetts, United States of America; 2 Department of Social Work, Ben-Gurion University of the Negev, Beer-Sheva, Israel; 3 School for Social Work, Smith College, Northampton, Massachusetts, United States of America; 4 Conflict Management and Resolution Program, Ben-Gurion University of the Negev, Beer-Sheva, Israel; 5 Department of Epidemiology, Harvard T. H. Chan School of Public Health, Boston, Massachusetts, United States of America; 6 Department of Psychiatry, Harvard Medical School, Boston, Massachusetts, United States of America; 7 Epidemiology Branch, Division of Intramural Population Health Research, Division of Intramural Research, Eunice Kennedy Shriver National Institute of Child Health and Human Development, Bethesda, Maryland, United States of America; 8 The Paul Baerwald School of Social Work and Social Welfare, The Hebrew University of Jerusalem, Jerusalem, Israel; 9 Department of Social Work, Zefat Academic College, Zefat, Israel; 10 Department of Community Mental Health, University of Haifa, Haifa, Israel; Sepuluh Nopember Institute of Technology: Institut Teknologi Sepuluh Nopember, INDONESIA

## Abstract

Mental Health Literacy (MHL) is pivotal for understanding and addressing mental disorders, often assessed using the comprehensive Mental Health Literacy Scale (MHLS) across six dimensions and 35 items. However, research on MHL within Arab populations is not just scarce, but alarmingly so, indicating a significant gap in knowledge regarding MHL in this demographic. This study aimed to fill this gap by adapting the MHLS into Arabic and evaluating its psychometric properties among three distinct Arabic-speaking groups: adolescents, students, and the general public. Data were collected using the translated MHLS, the General Health Questionnaire, and sociodemographic measures, employing a cross-sectional design. Validation procedures were conducted, including exploratory factor analysis, reliability testing, and face validity assessment. Construct validity was assessed using Pearson correlation analysis and t-tests. The validation process yielded three modified versions of the MHLS in Arabic: MHLS-Arabic-Adolescents (31 items, 5 factors), MHLS-Arabic-Students (30 items, 6 factors), and MHLS-Arabic-General Public (33 items, 5 factors). These tools demonstrated robust construct validity and high-reliability coefficients and stood as a testament to their adaptability and potential for use in diverse Arabic-speaking populations. This study suggests the nuanced nature of MHL and points to the importance of micro-context-specific validation. Providing three reliable MHLS Arabic versions could facilitate research on MHL among Arabic-speaking populations worldwide, potentially leading to tailored intervention initiatives that aim to enhance mental health outcomes and ensure equitable access to services within Arab communities globally.

## Introduction

Mental Health Literacy (MHL) was originally introduced by Jorm et al. [1] as the “knowledge and beliefs about mental disorders that facilitate their recognition, management, or prevention” (p. 182). It was conceived as an extension of health literacy, defined by the World Health Organization [2] as the attainment of knowledge, personal skills, and confidence necessary to improve personal and community health by modifying individual lifestyles and living conditions. Notably, Jorm [3] highlighted that health literacy traditionally focused on physical rather than mental health issues. Jorm’s MHL conceptual framework (2000) defines MHL as a multidimensional concept encompassing six dimensions:(1) Mental disorder recognition; (2) Risk factor knowledge; (3) Available professional help; (4) Information seeking knowledge; (5) Self-treatment knowledge; and (6) Attitudes that promote recognition or appropriate help-seeking behavior.

MHL has been recognized for its crucial role in enhancing mental health outcomes [3–6]. Research consistently highlights the association between MHL and mental health behaviors, such as intentions to seek help, actual help-seeking behaviors, engagement in health-promoting activities, and utilization of mental health services [7–9]. Consequently, MHL has become an important resource for promoting mental health, facilitating help-seeking, and addressing mental disorders [10].

While the concept of MHL has continuously evolved, Jorm’s [11] definition retains its prominence in the field, and the Mental Health Literacy Scale (MHLS) by O’Connor and Casey [12] stands out as the predominant tool for MHL assessment. The MHLS consists of 35 items that measure mental health knowledge, attitudes, and behaviors through the six dimensions of Jorm’s MHL framework [12]. The MHLS is pivotal in advancing MHL research, as evidenced by comparative analyses with alternative measurement tools highlighting its efficacy in assessing MHL and emphasizing its robustness and reliability [13]. Studies employing this scale have investigated MHL levels across diverse groups, including university students, Health care providers, individuals undergoing therapy, and the general public, showcasing its versatility across various populations [10,14–16].

Moreover, the scale has been translated and validated in multiple languages, such as French and Urdu, facilitating its application in different cultural contexts [17,18]. Recognizing that cultural nuances influence MHL [3,19], the scale’s translation and validation aim to ensure its appropriateness in diverse cultural settings. Despite these efforts, a notable gap exists in understanding MHL in non-Western and low-middle-income countries, where most studies using the MHLS have predominantly focused on Western samples [20].

One of the populations where insufficient attention has been devoted to MHL research is the Arab population. In a recent scoping review that relied on Jorm’s theoretical framework [3] and his definition of MHL, only nine studies were identified among Arabs. Seven of these studies employed a quantitative cross-sectional design, two utilizing the MHLS and five opting for an adapted version of the MHLS [10]. Moreover, it is widely recognized that there is a notable scarcity of local research in the mental health field in the Arab region, hindering the culturally appropriate development of diverse services in mental health care among Arabs [21]. Nonetheless, two recent studies have validated the MHLS tool [22,23]. While these studies hold significant relevance in the field, it’s worth noting that both were conducted within the specific context of Saudi Arabia, with the first study exclusively targeting female students. Furthermore, the absence of the Arabic version of the MHLS tool in these articles and elsewhere limits its potential contribution to advancing research on MHL within Arabs.

The absence of comprehensive mental health studies, particularly in the domain of MHL, is somewhat surprising given the substantial global Arab population of approximately 436 million. Although mental health data on Arabs is limited, the prevalence of mental disorders in the region is notably high [24]. For instance, recent meta-analyses indicate that the range of depression in Arabic countries spans from 3% to 50%, anxiety from 2% to 19%, and post-traumatic stress disorder from 2% to 36% [24]. Notably, Arabs tend to be less inclined to seek formal help. They are more prone to seek assistance for mental health issues from informal sources, such as religious counselors, friends, or family members, often relying on God as their primary path to healing [25,26]. A recent systematic review documented that mental health stigma served as a significant obstacle to seeking professional mental health assistance among Arabs [27]. The review underscores a common tendency among Arabs to attribute mental symptoms to supernatural causes, such as magic and the evil eye, rather than considering physiological or social factors. Mental health challenges are often perceived as a divine punishment or test [28,29]. Additionally, Arabs frequently hold highly negative perceptions of individuals facing mental health issues [25,27]. However, recent social and cultural changes among Arabs in the last few decades suggest a shift in knowledge and a transformation of stigmatizing beliefs about mental health [27,30].

### The current study

Due to the distinctive background of Arabs and their significant global presence, and considering that MHL is rooted in the individual’s context [3], this study endeavors to adapt the MHLS [12] into Arabic. The primary objective of the current study is to assess and validate the psychometric properties of the Arabic version of the MHLS among three Arabic-speaking groups within three different contexts: adolescents, emerging adults in academic settings (students), and individuals in the general public. Analyzing diverse groups within the same ethnic and cultural context stems from recognizing that MHL is linked to a more specific and concentrated context influenced by culture or language [10]. Evaluating the validity of questionnaires in Arabic across various samples ensures their accessibility and will contribute to advancing research in the realm of MHL among Arabs worldwide.

## Methods

### Research design

This cross-sectional study was part of three large research projects assessing MHL and mental health help-seeking among three different groups within the Arabic-speaking Palestinian citizens in Israel.

### Measures

#### Mental Health Literacy.

The Mental Health Literacy Scale (MHLS) [12] was used to evaluate participants’ MHL. Comprising 35 items, the self-report measure includes six dimensions:

1. *Ability to recognize disorders* (e.g., ‘To what extent do you think it is likely that Personality Disorders are a category of mental illness’).
2. *Knowledge of risk factors and causes* (e.g., ‘To what extent do you think it is likely that in general in Israel, men are MORE likely to experience an anxiety disorder compared to women’).
3. *Knowledge of self-treatment* (e.g., ‘To what extent do you think it would be helpful for someone to avoid all activities or situations that made them feel anxious if they were having difficulties managing their emotions’).
4. *Knowledge of professional help available* (e.g., ‘To what extent do you think it is likely that Cognitive Behaviour Therapy (CBT) is a therapy based on challenging negative thoughts and increasing helpful behaviors’).
5. *Knowledge of where to seek information* (e.g., ‘I am confident that I know where to seek information about mental illness’).
6. *Attitudes that promote recognition or appropriate help-seeking behavior* (e.g., ‘Seeing a mental health professional means you are not strong enough to manage your own difficulties’).

Items in dimensions (1), (2), and (4) were rated on a four-point Likert scale, ranging from 1 (very unlikely) to 4 (very likely). Items in dimension (3) were rated on a 4-point Likert scale, from 1 (very unhelpful) to 4 (very helpful). Lastly, items in dimensions (5) and (6) were rated on a 5-point Likert scale, from 1 (strongly disagree) to 5 (strongly agree). Twelve items were reverse-scored: items 10, 12, 15, and 20–28. Scores within each dimension were summed, with higher scores indicating a greater level of that particular aspect of MHL.

#### Psychological distress.

The General Health Questionnaire (GHQ-12) [31] was employed to evaluate psychological distress. Comprising 12 items, the questionnaire utilizes a 4-point Likert scale, which includes less than usual, no more than usual, more than usual, and much more than usual. For seven of these items, the scores were reversed. The questions prompt respondents to report recent feelings related to various variables. Examples include “Have you recently been thinking of yourself as worthless?”. A score of 0 is given for the first two choices and 1 for the next two. The scores of all items were aggregated to compute the overall index using the original scoring method, where a score of 0 is given for the first two choices and 1 for the next two. Scores on this scale range from 0 to 12, with higher values indicating elevated levels of psychological distress. The questionnaire was previously validated in Arabic [32].

#### Sociodemographic and clinical characteristics.

The sociodemographic questionnaire included questions about age, gender, subjective financial status (above average, average—indicative of the financial situation typical for most people in Israel, or below average; the adolescents and the students were asked to rate their families’ financial status), and self-reported history or current mental illness (Yes/No).

### Samples and procedure

This study involves three samples (adolescents, emerging adults in high academic settings- Students and adults from the general public) who met the specific criteria of being Arabic-speaking Palestinian citizens of Israel.

**The Adolescents sample** comprised 172 adolescents aged 14–18. Data was collected between March and July 2023. Research assistants utilized social media parent groups to recruit participants, sharing study details and contact information. Interested parents reached out to the principal researcher (first author). Those consenting to their children’s participation received a link containing study information and the questionnaire. Parents provided written consent, and adolescents completed the questionnaire independently. The comprehensive recruitment procedure is detailed in [33]. The Ethics Committee of Zefat Academic College approved this sample recruitment protocol.

**The Student sample** comprises 109 students enrolled at one of the higher education institutions in Israel (Universities and colleges affiliated with the Council for Higher Education in Israel). Data collection for this study occurred between March and July 2021. Two authors personally sent an internet link to potential participants, who were then asked to forward the link to other eligible participants using a snowball technique to enhance recruitment. The link led to a structured survey containing a study description and the researchers’ contact information. Before participation, all participants completed informed consent. The research protocol received approval from the Ethics Committee of Zefat Academic College. A detailed recruitment procedure is available in [34].

**The General Public sample** comprises 206 adults aged 18 and above. Data collection occurred between July and October 2021, with the recruitment protocol approved by the Human Subjects Ethics Committee of of Zefat Academic College. The first author conducted face-to-face interviews with potential participants and shared the survey form online with additional relevant individuals using the snowball technique. Both recruitment approaches included a study description, an informed consent form, and the authors’ contact details. Participation was contingent upon signing the form or indicating agreement. A detailed recruitment procedure is available in [10]

A convenience sample was employed in all three samples, meaning participants were recruited based on their availability and willingness to participate rather than through probability-based met. Participants completed a survey that included the MHLS, the GHQ-12, and the Sociodemographic and Clinical Characteristics questionnaire. All participants provided informed consent before undertaking the survey, which took approximately 20 minutes.

### Translation of the MHLS

The original Mental Health Literacy Scale (MHLS) was translated into Arabic and back-translated into English by two bilingual independent experts, following the recommendations of Sousa and Rojjanasrirat [35]. We performed this translation using Modern Standard Arabic, which is universally understood across the diverse accents and subcultures among Arabs in general. This approach ensures that the scale is applicable and accessible to Arabic-speaking populations globally.

Similar to the original scale, the translated version initially comprised 35 items, and no questions were omitted at this stage. The MHLS was pretested with 15 participants in each sample to ensure clarity and minor revisions were implemented based on their feedback. Importantly, no differences in wording were observed between the three groups, so the translated version remained consistent across all samples. The final version underwent review and approval by a panel of three Arab Palestinian mental health professionals, achieving face validation through consensus agreement on the translated Arabic version.

### Statistical analyses

All data were coded and analyzed using SPSS-25. (a) Descriptive analyses were used to summarize the characteristics of the participants and the scale, including means, standard deviations, and percentages. In addition, the assumptions of normal distribution for the MHLS items were tested, and the variables were found to fall within an acceptable range for normality among all samples (skewness: −1.38 to 0.23; kurtosis: −1.56 to 0.46). (b) Construct validity was assessed using exploratory factor analysis (EFA) with varimax rotation. Items with a rotated factor loading < 0.35 or exhibiting false loading inconsistent with theoretical understanding were eliminated. The EFA with varimax rotation was chosen to validate the tool in this study as it identifies the underlying factor structure of observed variables and simplifies the factors by maximizing the variance of squared loadings, ensuring they reflect the theoretical constructs being measured, as demonstrated in previous research validating the MHLS [22]. Spearman’s correlation coefficient was utilized to assess the association between variables within each obtained factor. (c) Cronbach’s alpha coefficients were calculated to determine the internal consistency of each factor. Values ranging from 0.70 to 0.80 were deemed good, those falling between 0.80 and 0.90 were categorized as very good, and scores exceeding 0.90 were regarded as excellent [36]. Also, corrected item-total correlations were computed to assess the strength of the relationship between each item and the overall score of the factor. Items with correlations below 0.20 were deemed non-contributory to the factor [37]. An analysis of floor and ceiling effects was conducted to assess the distribution of scores for each factor across different samples. No significant floor or ceiling effects were observed, as they did not exceed 15%, indicating that the scale factors were well-calibrated. (d) To evaluate Construct validity, Pearson correlation analysis, t-tests, or ANOVA were employed to investigate the relationship between the total score of each factor and participants’ characteristics in each sample, including age, gender, financial status, mental illness, and psychological distress. Homogeneity of variances for all variables was tested, and the results indicated that this assumption was satisfied. All p-values were two-tailed, and statistical significance was defined as p < 0.05. (e) Finally, following the completion of all analysis stages and the restructuring of the scale in each sample, which involved the removal of specific questions, a panel of five Palestinian Arab experts specializing in diverse populations relevant to the study reassessed the questionnaire as part of the face validity assessment. This expert team included researchers and mental health care providers from different professions and genders, all with considerable experience. To address missing data, we applied mean substitution, replacing missing values with the mean of each specific item calculated across all responses in the sample. The number of missing values was minimal, and there were no cases where more than 50% of the items for any respondent were unanswered.

## Results

### Description of the sample

Table 1 displays the characteristics of participants in each group sample. Most participants across groups were female, constituting 64% in the adolescents’ sample, 67.9% in the students’ sample, and 68.4% in the general public sample. The mean age was 16.25 years for the adolescent sample (range 14–18), 36.56 for the general public sample (range 18–72), and 25.13 for the student sample (range 19–40). In the sample of adolescents, 54.1% reported an above-average socioeconomic status, compared to 11.9% in the student sample and 24.8% in the general public sample. Furthermore, the majority of participants in all three samples reported no history or current diagnosis of mental illness, and the mean scores for psychological distress were low across all three groups.

**Table 1:** Sociodemographic and Clinical Characteristics of Participants in Each Group Sample.

	Adolescents sample (*N *= 172)	Students sample (*N *= 109)	General Public sample (*N *= 206)
**Age** *M* (*SD*)	16.25 (1.25)	25.13 (4.01)	36.56 (10.80)
**Gender** n (%)			
Male	62 (36)	35 (32.1)	65 (31.6)
Female	110 (64)	74 (67.9)	141 (68.4)
**Socioeconomic status** n **(%)**			
Above average	93 (54.1)	13 (11.9)	51 (24.8)
Average	70 (40.7)	43 (39.4)	88 (42.7)
Below average	9 (5.2)	53 (48.6)	67 (32.5)
**Mental Illness** n (%)			
Yes	12 (7.0)	15 (13.8)	16 (7.8)
No	160 (93.0)	94 (86.2)	190 (92.2)
**Psychological Distress** M (SD)	3.59 (2.72)	3.19 (1.80)	3.15 (2.64)

### Exploratory factor analysis

Tables 2, 3, and 4 present EFA results for adolescents, student samples, and adults from the general public community, respectively. These tables illustrate the final solution, including variables, factor loadings, corrected item-total correlations, alpha values with item deletion, and the Cronbach’s alpha of the subscale.

**Table 2:** Results of exploratory factor analysis and reliability tests of Mental Health Literacy Scale in the adolescents’ sample (N = 172); MHLS-Arabic-Adolescents.

	Mean (SD)	Factor loading	Reliability tests		
		**Corrected** **item-total** **correlation**	**Cronbach’s** **alpha if item** **is deleted**	**Overall** **Cronbach’s** **alpha**
I-1	2.70 (1.01)	0.69	0.62	0.53	
I-2	2.80 (1.02)	0.61	0.53	0.65	
I-3	2.94 (1.06)	0.72	0.48	0.71	
**Factor 1: Recognition of Anxiety and Depression Disorders**					**0.72**
I-4	2.83 (1.10)	0.55	0.62	0.81	
I-5	2.49 (1.08)	0.48	0.60	0.81	
I-6	2.07 (1.12)	0.60	0.60	0.81	
I-7	2.27 (1.12)	0.57	0.55	0.82	
I-8	2.73 (1.24)	0.65	0.58	0.82	
I-9	2.50 (1.13)	0.68	0.53	0.82	
I-11	2.73 (1.10)	0.60	0.45	0.83	
I-13	2.27 (1.18)	0.66	0.53	0.82	
I-14	2.52 (1.15)	0.52	0.47	0.83	
**Factor 2: Mental Health Knowledge**					**0.84**
I-16	3.07 (1.11)	0.77	0.63	0.73	
I-17	3.42 (1.13)	0.80	0.67	0.71	
I-18	3.01 (1.28)	0.67	0.42	0.84	
I-19	3.40 (1.13)	0.85	0.75	0.67	
**Factor 3: Knowledge of where to seek information**					**0.80**
I-21	4.13 (1.05)	0.55	0.45	0.75	
I-22	3.94 (1.16)	0.47	0.38	0.77	
I-25	3.26 (1.23)	0.66	0.52	0.74	
I-26	4.25 (0.99)	0.71	0.63	0.71	
I-27	3.75 (1.19)	0.76	0.56	0.72	
I-28	3.74 (0.96)	0.81	0.58	0.72	
**Factor 4: Attitude toward mental illness**					**0.77**
I-23	0.36 (1.01)	0.46	0.45	0.87	
I-24	3.98 (1.03)	0.35	0.36	0.87	
I-29	2.95 (0.97)	0.75	0.61	0.85	
I-30	3.41 (0.93)	0.81	0.74	0.84	
I-31	3.56 (0.94)	0.81	0.77	0.84	
I-32	3.45 (0.94)	0.80	0.73	0.84	
I-33	3.37 (1.07)	0.71	0.62	0.85	
I-34	2.73 (1.13)	0.76	0.57	0.86	
I-35	3.21 (0.97)	0.74	0.64	0.85	
**Factor 5: Attitudes towards people with a mental** **health problem**					**0.87**

**Table 3:** Results of exploratory factor analysis and reliability tests of Mental Health Literacy Scale in the students sample (N = 109); MHLS-Arabic-Students.

	Mean (SD)	Factor loading	Reliability tests		
			**Corrected** **item-total** **correlation**	**Cronbach’s** **alpha if item** **is deleted**	**Overall** **Cronbach’s** **alpha**
I-1	2.76 (1.02)	0.48	0.67	0.84	
I-2	2.89 (0.98)	0.41	0.63	0.85	
I-4	3.01 (0.96)	0.81	0.63	0.85	
I-5	3.07 (0.89)	0.76	0.49	0.86	
I-6	2.72 (1.15)	0.72	0.67	0.85	
I-7	3.01 (1.12)	0.65	0.72	0.84	
I-8	3.21 (0.94)	0.73	0.70	.84	
**Factor 1: Ability to recognize disorders**					**0.87**
I-9	2.85 (1.17)	0.80	0.51	0.72	
I-11	2.90 (1.00)	0.74	0.55	0.69	
I-13	2.92 (1.07)	0.47	0.60	0.66	
I-14	3.00 (1.01)	0.58	0.53	0.70	
**Factor 2: Mental health Knowledge**					**0.75**
I-16	3.66 (0.81)	0.81	0.59	0.74	
I-17	3.93 (0.88)	0.71	0.58	0.74	
I-18	3.73 (0.87)	0.63	0.57	0.75	
I-19	3.83 (0.88)	0.78	0.63	0.71	
**Factor 3: Knowledge of where to seek information**					**0.79**
I-21	4.08 (1.02)	0.63	0.60	0.80	
I-22	3.95 (1.07)	0.83	0.55	0.82	
I-23	3.60 (1.12)	0.72	0.68	0.76	
I-24	3.93 (1.15)	0.77	0.75	0.72	
**Factor 4: Attitude toward mental illness**					**0.83**
I-25	3.66 (1.15)	0.53	0.60	0.84	
I-26	4.12 (0.96)	0.38	0.70	0.80	
I-27	3.99 (1.12)	0.67	0.74	0.77	
I-28	3.91 (0.98)	0.78	0.69	0.80	
**Factor 5: Attitudes about Mental health treatment**					**0.84**
I-29	3.09 (0.94)	0.51	0.60	0.89	
I-30	3.65 (0.94)	0.79	0.78	0.87	
I-31	3.58 (1.01)	0.82	0.79	0.87	
I-32	3.61 (0.88)	0.88	0.76	0.88	
I-33	3.10 (1.17)	0.76	0.74	0.88	
I-34	2.67 (1.17)	0.55	0.62	0.89	
I-35	3.28 (0.95)	0.79	0.67	0.88	
**Factor 6: Attitudes towards people with a mental health problem**					**0.90**

**Table 4:** Results of exploratory factor analysis and reliability tests of Mental Health Literacy Scale in the general public sample (N = 206); MHLS-Arabic-General Public.

	Mean (SD)	Factor loading	Reliability tests		
			**Corrected** **item-total** **correlation**	**Cronbach’s** **alpha if item** **is deleted**	**Overall** **Cronbach’s** **alpha**
I-1	2.57 (0.98)	0.64	0.60	0.91	
I-2	2.87 (0.98)	0.81	0.72	0.91	
I-3	2.82 (1.00)	0.78	0.72	0.91	
I-4	2.76 (1.04)	0.81	0.77	0.91	
I-5	2.78 (1.03)	0.82	0.78	0.91	
I-6	2.46 (1.08)	0.72	0.69	0.91	
I-7	2.45 (1.11)	0.66	0.68	0.91	
I-8	2.85 (1.05)	0.75	0.74	0.91	
I-9	2.76 (1.03)	0.63	0.64	0.91	
I-10	2.30 (0.98)	0.53	0.51	0.92	
I-13	2.84 (1.10)	0.52	0.65	0.91	
I-14	2.83 (1.17)	0.38	0.51	0.92	
**Factor 1: Mental health Knowledge**					**0.92**
I-11	2.63 (0.99)	0.79			
I-12	2.58 (1.01)	0.76			
**Factor 2: Knowledge of self-treatment**					**r = 0.53 (p < 0.001)**
I-16	3.54 (1.18)	0.82	0.80	0.87	
I-17	3.76 (1.16)	0.73	0.78	0.88	
I-18	3.71 (1.23)	0.80	0.73	0.90	
I-19	3.84 (1.12)	0.75	0.85	0.86	
**Factor 3: Knowledge of where to seek information**					**0.91**
I-21	3.99 (0.98)	0.50	0.62	0.79	
I-22	4.00 (1.12)	0.40	0.55	0.80	
I-25	3.58 (1.07)	0.71	0.51	0.81	
I-26	4.08 (0.93)	0.77	0.65	0.78	
I-27	4.02 (0.94)	0.76	0.70	0.77	
I-28	3.87 (0.91)	0.65	0.55	0.80	
**Factor 4: Attitude toward mental illness**					**0.82**
I-23	3.79 (0.88)	0.38	0.57	0.91	
I-24	3.89 (0.96)	0.38	0.52	0.91	
I-29	2.89 (0.98)	0.81	0.73	0.90	
I-30	3.26 (0.94)	0.88	0.81	0.90	
I-31	3.36 (0.89)	0.85	0.81	0.90	
I-32	3.25 (0.89)	0.87	0.82	0.89	
I-33	2.80 (0.96)	0.74	0.73	0.90	
I-34	2.73 (1.07)	0.65	0.62	0.91	
I-35	3.04 (0.97)	0.75	0.73	0.90	
**Factor 5: Attitudes towards people with a mental health problem**					**0.91**

#### The Adolescents’ sample.

In the adolescent sample, four items from the original MHLS were excluded (items 10, 12, 15, and 20) due to either false loading inconsistent with theoretical understanding or a low factor loading of less than 0.35. The final model comprised 31 items and was divided into five factors instead of six in the original MHLS. As depicted in Table 2, Factor 1, “Recognition of Anxiety and Depression Disorders,” demonstrated a Cronbach’s alpha of 0.72 with individual corrected item-total correlations ranging between 0.48 and 0.62. The Spearman correlation between the three items of Factor 1 was significant (p < 0.001) and ranged between r = 0.32 and r = 0.57. Factor 2, “Mental Health Knowledge,” had a Cronbach’s alpha of 0.84 with individual corrected item-total correlations between 0.45 and 0.62. The Spearman correlations between the nine items of Factor 2 were significant (p < 0.05) and ranged between r = 0.17 and r = 0.56. Factor 3, “Knowledge of where to seek information,” remained consistent with the original MHLS, comprising the same four items and demonstrating a Cronbach’s alpha of 0.80. Individual corrected item-total correlations ranged between 0.67 and 0.85, with a significant Spearman correlation between the items (p < 0.001) ranging between r = 0.67 and r = 0.85. The last dimension in the original MHLS, “Attitudes that promote recognition or appropriate help-seeking behavior,” was divided into two factors in the adolescent sample. Factor 4, “Attitude toward mental illness,” displayed a Cronbach’s alpha of 0.77 with individual corrected item-total correlations ranging between 0.38 and 0.63. The Spearman correlation between the six items of this factor was significant (p < 0.001) and ranged between r = 0.16 and r = 0.57. In parallel, Factor 5, “Attitudes towards people with a mental health problem,” had a Cronbach’s alpha of 0.87 with individual corrected item-total correlations between 0.36 and 0.77. The Spearman correlation between the nine items of this factor was significant (p < 0.01) and ranged between r = 0.22 and r = 0.73.

#### The student sample.

In the student sample, five items were excluded (items 3, 10, 12, 15, and 20). The final model comprised 30 items and was divided into six factors. As illustrated in Table 3 Factor 1, ‘Ability to Recognize Disorders,’ remained similar to dimension 1 in the original MHLS, excluding item 3. This factor exhibited a Cronbach’s alpha of 0.87, with individual corrected item-total correlations ranging between 0.49 and 0.72. The Spearman correlation among the three items of Factor 1 was significant (p < 0.001) and ranged between r = 0.42 and r = 0.63. Factor 2, “Mental Health Knowledge,” was reduced to four items compared to the same factor in the previous samples. It combines items from the dimensions ‘Knowledge of Risk Factors and Causes,’ ‘Knowledge of Professional Help Available,’ and ‘Knowledge of Self-Treatment’ in the original MHLS. This factor had a Cronbach’s alpha of 0.75, with individual corrected item-total correlations between 0.51 and 0.60. The Spearman correlation among the nine items of Factor 2 was significant (p < 0.001) and ranged between r = 0.35 and r = 0.64. Factor 3, “Knowledge of Where to Seek Information,” remained consistent with the original MHLS and factor 3 in the adolescent and general public samples, including the same four items. It demonstrated a Cronbach’s alpha of 0.79, with individual corrected item-total correlations ranging between 0.57 and 0.63. The Spearman correlation among the items was significant (p < 0.001), ranging between r = 0.40 and r = 0.65. The last dimension in the original MHLS, “Attitudes that Promote Recognition or Appropriate Help-Seeking Behavior,” was divided into three factors in the student sample. Factor 4, “Attitude toward mental illness” displayed a Cronbach’s alpha of 0.83, with individual corrected item-total correlations ranging between 0.55 and 0.75. The Spearman correlation among the four items of this factor was significant (p < 0.001) and ranged between r = 0.46 and r = 0.74. Factor 5, “Attitudes about Mental health treatment,” had a Cronbach’s alpha of 0.84, with individual corrected item-total correlations between 0.60 and 0.74. The Spearman correlation among the four items of this factor was significant (p < 0.001) and ranged between r = 0.45 and r = 0.66. Lastly, Factor 6, “Attitudes Towards People with a Mental Health Problem,” had a Cronbach’s alpha of 0.90, with individual corrected item-total correlations between 0.60 and 0.79. The Spearman correlation among the seven items of this factor was significant (p < 0.001) and ranged between r = 0.32 and r = 0.87.

#### The general public sample.

In the general population sample, two items (items 15 and 20) were excluded for reasons consistent with those in the previous sample. The final model comprised 33 items and was organized into five factors, a departure from the original six factors in the MHLS.

As depicted in Table 4, Factor 1, “Mental Health Knowledge,” exhibited a robust Cronbach’s alpha of 0.92, with individual corrected item-total correlations ranging between 0.51 and 0.78. The Spearman correlation among the 12 items of Factor 1 was significant (p < 0.001), ranging between r = 0.24 and r = 0.74. This factor amalgamated the three dimensions of ‘Ability to recognize disorders,’ ‘Knowledge of risk factors and causes,’ and ‘Knowledge of professional help available’ from the original MHLS. Factor 2, ‘Knowledge of Self-Treatment,’ retained its structure from the original MHLS but with two items instead of three. The Spearman correlation between the items was significant, r = 0.53 (p < 0.001). The other three factors mirrored those in the adolescent sample, maintaining the same items and division. Factor 3, “Knowledge of Where to Seek Information,” remained consistent with the original MHLS, boasting a Cronbach’s alpha of 0.91. Individual corrected item-total correlations ranged between 0.73 and 0.85, with a significant Spearman correlation between the items (p < 0.001) ranging between r = 0.59 and r = 0.81. Factor 4, “Attitude Toward Mental Illness,” demonstrated a Cronbach’s alpha of 0.82, with individual corrected item-total correlations ranging between 0.51 and 0.70. The Spearman correlation between the six items of this factor was significant (p < 0.001), ranging between r = 0.28 and r = 0.63. Factor 5, “Attitudes Towards People with a Mental Health Problem,” displayed a Cronbach’s alpha of 0.91, with individual corrected item-total correlations between 0.52 and 0.82. The Spearman correlation among the nine items of this factor was significant (p < 0.001), ranging between r = 0.30 and r = 0.82 (Table 4).

### Concurrent validity

Table 5 presents the new MHL factors’ means, standard deviations, and correlations among the adolescents, student samples, and adults from the general public. It is evident that moderate to high levels of MHL are observed in all factors among the three distinct samples.

**Table 5:** Means, Standard Deviations, and correlates for MHL factors by group samples.

	Possible Range	*M (SD)*	*Age*	*Gender*	Financial status	Mental Illness	Psychological Distress
**MHL factors in the adolescents’ sample**							
Factor 1: Recognition of Anxiety and Depression Disorders	3-12	8.44 (2.49)	NS	NS	F_(2,169)_ = 4.49*	NS	r = 0.24**
Factor 2: Mental Health Knowledge	9-36	22.26 (6.87)	NS	NS	NS	NS	NS
Factor 3: Knowledge of where to seek information	4-20	12.69 (3.87)	NS	NS	NS	NS	r = −0.19*
Factor 4: Attitude toward mental illness	6-30	23.02 (4.56)	NS	NS	NS	NS	r = −0.17*
Factor 5: Attitudes towards people with a mental health problem	6-45	29.95 (6.61)	NS	NS	NS	t_(166)_ = −1.99*	NS
**MHL factors in the students’ sample**							
Factor 1: Ability to recognize disorders’	8-32	23.35 (5.73)	NS	t_(107)_ = −2.70**	NS	NS	NS
Factor 2: Mental health Knowledge	4-16	11.55 (3.25)	NS	t_(107)_ = −4.32***	NS	NS	r = 0.19*
Factor 3: Knowledge of where to seek information	4-20	15.10 (2.66)	NS	t_(107)_ = −3.35**	NS	NS	NS
Factor 4: Attitude toward mental illness	4-20	15.43 (3.68)	NS	t_(50.94)_ = −4.22***	NS	NS	NS
Factor 5: Attitudes about Mental health treatment	4-20	15.46 (3.66)	NS	t_(107)_ = −5.36***	F_(2,106)_ = 3.46*	NS	NS
Factor 6: Attitudes towards people with a mental health problem	7-35	22.87 (5.64)	NS	NS	NS	NS	NS
**MHL factors in the general public sample**							
Factor 1: Mental health Knowledge	12-48	31.67 (9.25)	r = −0.26***	t_(109.08)_ = −4.76***	F_(2,203)_ = 3.09*	NS	NS
Factor 2: Knowledge of self-treatment	1-8	5.18 (1.76)	r = −0.15**	t_(202)_ = −2.39*	NS	NS	NS
Factor 3: Knowledge of where to seek information	4-20	14.80 (4.20)	NS	t_(106.36)_ = −4.51***	F_(2,200)_ = 4.97**	NS	NS
Factor 4: Attitude toward mental illness	6-30	23.48 (4.36)	r = −0.21**	t_(200)_ = −4.01***	F_(2,199)_ = 4.57*	NS	r = 0.24**
Factor 5: Attitudes towards people with a mental health problem	6-45	28.61 (6.81)	r = −0.22**	t_(201)_ = −3.61***	NS	t_(201)_ = 2.90*	r = 0.34***

*p < .05, **p < .01, ***p < .001

Concerning the correlations, as depicted in Table 5, different factors exhibit varying associations with participant characteristics in the three samples. Among adolescents, we found significant differences in Recognition of Anxiety and Depression Disorders among participants with different levels of socioeconomic status. Individuals with an average and above socioeconomic status demonstrate higher MHL in Recognizing Anxiety and Depression Disorders compared to those below average. Moreover, individuals with a current or history of mental illness exhibit more positiveAttitudes towards people with mental health problems. While a positive significant correlation exists between psychological distress and Recognition of Anxiety and Depression Disorders, negative significant correlations are found between psychological distress and Knowledge of where to seek information, as well as Attitude toward mental illness.

Among the student sample, female students report significantly higher MHL in all factors except Attitudes toward people with mental health problems. Those with an average and above socioeconomic status demonstrate significantly higher MHL only in the factor Attitudes about Mental health treatment. Psychological distress is positively and significantly correlated only with the MHL factor Mental Health Knowledge.

Finally, among adults from the general public sample, age is positively and significantly related to all MHL factors except Knowledge of where to seek information. At the same time, women significantly display higher MHL in all aspects. Those with an average and above socioeconomic status demonstrate significantly higher MHL in Mental Health Knowledge, Knowledge of where to seek information and Attitude toward mental illness. Similar to the adolescent sample, individuals with a history of mental illness exhibit more positive attitudes towards people with mental health problems. Positive significant correlations are found between psychological distress and Attitude toward mental illness, as well as Attitudes toward people with a mental health problem.

### Face validity

The questionnaire underwent a rigorous evaluation process by experts, as detailed in the statistical analysis subsection, where each factor and its assigned items were thoroughly assessed through the EFA. An exceptional level of agreement was achieved, with experts expressing high agreement rates ranging from 80% to 100% in all items and in each sample. This significant level of inter-expert agreement is a strong indicator of face validity.

Table 6 consolidates the findings, displaying items in English and Arabic and the association of each item with its corresponding factor as determined by the analysis within each research sample.

**Table 6:** English and Arabic Items and Their Division into Factors Across Samples.

Items in English	Items in Arabic	Adolescents’ Item Distribution	Students’ Item Distribution	The general public’s Item Distribution
**Item #1:** If someone became extremely nervous or anxious in one or more situations with other people (e.g., a party) or performance situations (e.g., presenting at a meeting) in which they were afraid of being evaluated by others and that they would act in a way that was humiliating or feel embarrassed, then to what extent do you think it is likely they have **Social Phobia**	عندما يصبح شخص ما عصبياً أو قلقاً جداً في مواقف اجتماعية معينة، مثل الحفلات، أو خلال أداء معين مثل العروض في الاجتماعات، ويخشى أن يتم تقديره بشكل سلبي من قبل الآخرين الذين قد يكونون مهينين أو محرجين، إلى أي مدى تعتقد أنه من المحتمل أن يعاني من **الرهاب الاجتماعي**	Factor 1	Factor 1	Factor 1
**Item #2:** If someone experienced excessive worry about a number of events or activities where this level of concern was not warranted, had difficulty controlling this worry and had physical symptoms such as having tense muscles and feeling fatigued then to what extent do you think it is likely they have **Generalised Anxiety Disorder**	إذا شعر شخص ما بالقلق الشديد بشأن عدة أحداث أو نشاطات، حيث يكون هذا القلق غير مبرر، ويواجه صعوبة في التحكم به، مع الاحساس بأعراض جسدية مثل توتر العضلات والإرهاق، فما هو الاحتمال بالنسبة لهذا الشخص بأن يعاني **من اضطراب القلق العام**	Factor 1	Factor 1	Factor 1
**Item #3:** If someone experienced a low mood for two or more weeks, had a loss of pleasure or interest in their normal activities and experienced changes in their appetite and sleep then to what extent do you think it is likely they have **Major Depressive Disorder**	إذا شعر شخص ما بمزاج سيء لمدة أسبوعين أو أكثر، بالإضافة إلى عدم الاستمتاع بالأنشطة الروتينية وفقدان الاهتمام بها، وظهور تغييرات في الشهية ونمط النوم، إلى أي مدى، حسب رأيك، يعاني هذا الشخص من **اضطراب الاكتئاب الشديد**	Factor 1	Excluded	Factor 1
**Item #4**: To what extent do you think it is likely that **Personality Disorders** are a category of mental illness	**إلى أية مدى** تعتقد أن **اضطرابات الشخصية** قد تكون نوعا من الأمراض النفسية	Factor 2	Factor 1	Factor 1
**Item #5:** To what extent do you think it is likely that **Dysthymia** is a disorder	إلى أي مدى تعتقد أن **الاكتئاب الجزئي** قد يُعتبر اضطرابًا	Factor 2	Factor 1	Factor 1
**Item#6:** To what extent do you think it is likely that the diagnosis of **Agoraphobia** includes anxiety about situations where escape may be difficult or embarrassing	إلى أي مدى تعتقد أن تشخيص **رُهاب الخلاء** قد يتضمن الشعور بالقلق من المواقف التي يكون فيها الهرب صعبًا أو محرجًا	Factor 2	Factor 1	Factor 1
**Item #7:** To what extent do you think it is likely that the diagnosis of **Bipolar Disorder** includes experiencing periods of elevated (i.e., high) and periods of depressed (i.e., low) mood	إلى أي مدى تعتقد أن تشخيص **اضطراب ثنائي القطب** قد يتضمن فترات من الشعور بالبهجة (مزاج عال) وفترات من الشعور بالكآبة (مزاج منخفض)	Factor 2	Factor 1	Factor 1
**Item #8:** To what extent do you think it is likely that the diagnosis of **Drug Dependence** includes physical and psychological tolerance of the drug (i.e., require more of the drug to get the same effect)	إلى أي مدى تعتقد أن تشخيص **الإدمان على المخدرات** قد يشمل التعلق النفسي والجسدي بالمخدرات (أي، الحاجة إلى كميات أكبر من المخدرات لتحقيق نفس التأثير)	Factor 2	Factor 1	Factor 1
**Item #9:** To what extent do you think it is likely that in general among Arabs, **women are MORE likely to experience a mental illness of any kind compared to men**	إلى أي مدى تعتقد أن **النساء العربيات بشكل عام قد يكون لديهن ميلاً أكبر لتعاني من أضطرابات نفسية متنوعة مقارنة بالرجال**	Factor 2	Factor 2	Factor 1
**Item #10:** To what extent do you think it is likely that in general among Arabs**, men are MORE likely to experience an anxiety disorder compared to women**	إلى أي مدى تعتقد أن **الرجال العرب بشكل عام قد يكونون أكثر عرضة لتجارب اضطرابات القلق مقارنة بالنساء**	Excluded	Excluded	Factor 1
**Item #11:** To what extent do you think it would be helpful for someone to **improve their quality of sleep** if they were having difficulties managing their emotions (e.g., becoming very anxious or depressed)	إلى أي مدى تعتقد **أن تحسين جودة النوم** يمكن أن يكون ناجعا لشخص يعاني من صعوبات في التحكم بمشاعره، مثل الغضب أو الإحباط الشديد	Factor 2	Factor 2	Factor 2
**Item #12:** To what extent do you think it would be helpful for someone to **avoid all activities or situations that made them feel anxious** if they were having difficulties managing their emotions	إلى أي مدى تعتقد أن **تجنب حالة أو نشاط يسببان الشعور بالقلق** قد يكون ناجعا لشخص يعاني من صعوبات في السيطرة على مشاعره	Excluded	Excluded	Factor 2
**Item #13:** To what extent do you think it is likely that **Cognitive Behaviour Therapy (CBT)** is a therapy based on challenging negative thoughts and increasing helpful behaviors	إلى أي مدى تعتقد أن **العلاج الإدراكي السلوكي CBT** قد يكون علاجًا يعتمد على تحدي الأفكار السلبية وتعزيز السلوكيات المساعدة	Factor 2	Factor 2	Factor 1
**Item #14:** Mental health professionals are bound by confidentiality; however there are certain conditions under which this does not apply. To what extent do you think it is likely that the following is a condition that would allow a mental health professional to **break confidentiality**: *If you are at immediate risk of harm to yourself or others*	واجب على الاختصاصيين العاملين في مجال الصحة النفسية الحفاظ على السرية، ومع ذلك، هناك حالات تستدعي كسر هذا الالتزام. إلى أي مدى تعتقد أنه من المحتمل أن تسمح الحالة التالية لاخصائي صحة نفسية بالكشف عن المعلومات المحفوظة سراً: **في حالة كونك تشكل خطرًا فوريًا على نفسك أو على الآخرين**	Factor 2	Factor 2	Factor 1
**Item #15:** To what extent do you think it is likely that the following is a condition that would allow a mental health professional to **break confidentiality**: *if your problem is not life-threatening and they want to assist others to better support you*	**واجب على الاختصاصيين العاملين في مجال الصحة النفسية الحفاظ على السرية، ومع ذلك، هناك حالات تستدعي كسر هذا الالتزام.** إلى أي مدى تعتقد أنه من المحتمل أن تسمح الحالة التالية لاخصائي صحة نفسية بالكشف عن المعلومات المحفوظة سراً: **إذا لم تكن مشكلتك تشكل خطرًا على الحياة، ولكنه يرغب في مساعدة الآخرين على دعمك بشكل أفضل**	Excluded	Excluded	Excluded
**Item #16:** I am confident that I know where to seek information about mental illness	أنا متأكد من أني أعرف أين أجد المعلومات حول الاضطرابات النفسية	Factor 3	Factor 3	Factor 3
**Item #17**: I am confident using the computer or telephone to seek information about mental illness	أشعر بالثقة في قدرتي على استخدام الحاسوب أو الهاتف الجوال للوصول إلى معلومات حول الاضطرابات النفسية	Factor 3	Factor 3	Factor 3
**Item #18:** I am confident attending face to face appointments to seek information about mental illness(e.g., seeing the GP)	أشعر بالثقة في إجراء لقاء مباشر، مثل لقاء مع طبيب العائلة الخاص بي، للحصول على معلومات حول الاضطرابات النفسية	Factor 3	Factor 3	Factor 3
**Item #19:** I am confident I have access to resources (e.g., GP, internet, friends) that I can use to seek information about mental illness	أشعر بالثقة في قدرتي على الوصول إلى مصادر متنوعة مثل الإنترنت، والأصدقاء، وطبيب العائلة، التي يمكنني استخدامها للبحث عن معلومات حول الاضطرابات النفسية	Factor 3	Factor 3	Factor 3
**Item #20:** People with a mental illness could snap out if it if they wanted	يمكن للأشخاص الذين يعانون من اضطرابات نفسية أن يتغلبوا عليها إذا رغبوا في ذلك	Excluded	Excluded	Excluded
**Item #21:** A mental illness is a sign of personal weakness	الاضطرابات النفسية **هي دليلا على ضعف شخصي**	Factor 4	Factor 4	Factor 4
**Item #22:** A mental illness is not a real medical illness	الاضطرابات النفسية ليست أمراض حقيقية في الواقع	Factor 4	Factor 4	Factor 4
**Item #23:** People with a mental illness are dangerous	الأشخاص الذين يعانون من اضطرابات نفسية **هم أشخاص خطرون**	Factor 5	Factor 4	Factor 5
**Item #24:** It is best to avoid people with a mental illness so that you don’t develop this problem	من الأفضل تجنب التعامل مع الأشخاص الذين يعانون من اضطرابات نفسية لتفادي تطوير مشاكل مثل هذه	Factor 5	Factor 4	Factor 5
**Item #25:** If I had a mental illness I would not tell anyone	إذا كنت أعاني من اضطراب نفسي، لن أخبر أحدًا بها	Factor 4	Factor 5	Factor 4
**Item #26:** Seeing a mental health professional means you are not strong enough to manage your own difficulties	اجراء اللقاءات مع مختصين في مجال الصحة النفسية تعني أنك لست قويًا بما يكفي لإدارة صعوباتك بمفردك	Factor 4	Factor 5	Factor 4
**Item #27:** If I had a mental illness, I would not seek help from a mental health professional	إذا كنت أعاني من اضطراب نفسي، فلن ألجأ إلى مساعدة من مختصين في مجال الصحة النفسية	Factor 4	Factor 5	Factor 4
**Item #28:** I believe treatment for amental illness, provided by a mental health professional, would not be effective.	أؤمن بأن العلاج الذي يُقدمه مختصون في مجال الصحة النفسية ليس ناجعا في علاج الاضطرابات النفسية	Factor 4	Factor 5	Factor 4
**Item #29:** How willing would you be to move next door to someone with a mental illness?	إلى أي مدى توافق على الانتقال للعيش بالقرب من شخص يعاني من اضطراب نفسي	Factor 5	Factor 6	Factor 5
**Item #30:** How willing would you be to spend an evening socialising with someone with a mental illness?	إلى أي مدى توافق على قضاء أمسية ترفيهية مع شخص يعاني من اضطراب نفسي	Factor 5	Factor 6	Factor 5
**Item #31:** How willing would you be to make friends with someone with a mental illness?	إلى أي مدى توافق على تكوين صداقة مع شخص يعاني من مرض نفسي	Factor 5	Factor 6	Factor 5
**Item #32:** How willing would you be to have someone with a mental illness start working closely with you on a job?	إلى أي مدى توافق على أن يبدأ شخص يعاني من اضطراب نفسي بالعمل معك عن كثب على مهمة محددة	Factor 5	Factor 6	Factor 5
**Item #33:** How willing would you be to have someone with a mental illness marry into your family?	إلى أي مدى توافق على أن ينضم شخص يعاني من اضطراب نفسي إلى عائلتك	Factor 5	Factor 6	Factor 5
**Item #34:** How willing would you be to vote for a politician if you knew he had suffered a mental illness?	إلى أي مدى توافق على التصويت لسياسي، إذا كنت تعرف أن لديه اضطراب نفسي	Factor 5	Factor 6	Factor 5
**Item #35:** How willing would you be to employ someone if you knew they had a mental illness?	إلى أي مدى توافق على توظيف شخص، إذا كنت تعرف أن لديه اضطراب نفسي	Factor 5	Factor 6	Factor 5

## Discussion

This study aimed to evaluate the reliability and validity of the MHLS [12] in Arabic among three distinct groups: adolescents, emerging adults in academic settings (students), and individuals in the general public. The evaluation of the original MHLS among these groups (MHLS-Arabic-Adolescents, MHLS-Arabic-General Public, and MHLS-Arabic-Students) demonstrated strong construct validity and higher reliability coefficients compared to other published studies, both in general and particularly those conducted among Arab participants [9,17,18,22,23].

However, our analyses raise several significant and intriguing points for discussion. First, our empirical findings not only corroborate the multidimensional concept of MHL [3], but also hint at the possibility that MHL is influenced by more nuanced contexts beyond mere cultural and linguistic factors. This suggests a more profound, more complex interplay that warrants further exploration. This observation is underscored by the different divisions of the MHLS dimensions among three groups originating from the same cultural and societal background and sharing the same language and dialect. Our findings underscore the significance of incorporating micro-contexts into the validation of the MHLS. Validation measures for MHL tools must be tailored to the specific nuanced to adequately address the complexities of mental health understanding and management across diverse settings. This understanding allows for ongoing research to explore aspects requiring deeper comprehension, thereby facilitating the development of more focused intervention programs grounded in these studies.

Second, it was interesting to observe that items 15, “A mental health professional can break confidentiality if your problem is not life-threatening and they want to assist others to better support you,” and 20, “People with a mental illness could snap out of it if they wanted,” received low loading factors across all three samples and as a result were excluded. This finding is consistent with previous research that included various MHL versions. These two items were omitted in Saudi Arabic, French, Chinese, and Slovenian versions [16,17,22,23,36,38]. It is plausible that item I-15 lacks clarity, as typically, involving family members in shared decision-making regarding the treatment and management of mental disorders is an important aspect of mental health care [39,40]. Regarding the unclarity of item I-20, Wang et al. [16] argue that the statement appears overly absolute. They suggest that mental illness could be self-cured in certain instances, such as through self-practiced mindfulness practices [41]. These findings highlight the necessity for continual refinement and validation of assessment tools to ensure accurate measurement of MHL across diverse populations.

Thirdly, within the MHLS, the original dimension, “Attitudes that promote recognition or appropriate help-seeking behavior,” underwent differentiation into two factors among adolescents and the general public: “Attitude toward mental illness” and “Attitudes towards people with a mental health problem.” However, among the student sample, it further subdivided into three factors: “ Attitude toward mental illness”, “Attitudes about Mental health treatment,” and “Attitudes towards people with a mental health problem.” These findings echo previous studies, which also observed the division of this dimension into multiple attitudes [17,18,42]. The differentiation of the original dimension in the MHLS into distinct factors among different populations underscores the intricate nature of attitudes toward mental health and help-seeking behaviors. Our research underscores the importance of differentiating attitudes toward mental illness, which span a broader spectrum encompassing risk factors, treatments, and additional factors, as opposed to attitudes solely targeting individuals with mental disorders.

Lastly, to our knowledge, this study is the first to examine the validity of the MHLS tool among adolescents. This is notable due to the existing research gap in MHL studies focusing on this population [43,44], compounded by a scarcity of instruments tailored for assessing MHL within this age group [45]. The findings of the study, particularly the identification of a unique factor related to recognizing depression and anxiety disorders, highlight the importance of conceptualizing and measuring MHL with adjustments tailored to adolescent populations. The inclusion of a factor associated with depression and anxiety recognition is unsurprising, considering the World Health Organization’s assertation that these conditions are the primary contributors to disability among adolescents [46]. This underscores the urgency for intervention programs to prioritize MHL efforts focused on the prevention of depression and anxiety among adolescents in this vulnerable group.

### Limitations and future research

Two limitations of the study should be noted. First, the study was specifically conducted among the Arabic-speaking Palestinian citizens of Israel. Given the diversity among Arab populations worldwide, concentrating on this specific subgroup could potentially affect the generalizability of the MHLS validation. While the use of modern standard literary Arabic, widely accepted and employed among Arabs universally, may help mitigate the potential impact of this limitation, language alone may not capture the full range of cultural and contextual factors that shape MHL. Variables such as national health systems, religious norms, conflict exposure, and migration histories may all influence how individuals understand and respond to MHLS items, even when the language is standardized. These factors could affect the interpretation, relevance, and validity of certain items across diverse Arab contexts.

We recommend that future studies examine our adapted version among other Arab populations worldwide to assess its applicability and effectiveness in varying contexts. This broader examination will enhance the generalizability of our findings and provide valuable insights into the nuances of MHL within different Arab communities.

Second, data collection relied on self-reports, potentially introducing inaccuracies in participant responses and potentially amplifying social desirability bias. Additionally, in our factor analysis, Factor 2 within the general public sample consists of only two items, which may not offer sufficient robustness and may reflect a sample-specific pattern or item-level variation rather than a stable construct. While we followed previous studies validating the MHLS in our statistical analysis to ensure that the factors reflect the theoretical constructs of mental health literacy [22], we also drew on literature suggesting that EFA is sufficient for the cross-cultural adaptation of health-related scales [47,48]. However, some aspects of validity and reliability were not assessed, such as confirmatory factor analysis and test-retest reliability, as the study was conducted at a single point in time. Importantly, convergent and discriminant validity were not tested using external measures, which limits the strength of our psychometric conclusions’ strength. Future studies should employ diverse data collection methods to triangulate information and enhance the reliability of MHL tools validation, but also incorporate external validation tools, and investigate the factor structure further, particularly in the general public version of the scale, to refine the instrument and enhance its cross-contextual utility. Integrating our quantitative findings with qualitative studies can offer a more comprehensive understanding of the MHLS and help mitigate the impact of potential inaccuracies and social desirability bias.

## Conclusion and implications

In conclusion, this study presents three modified MHLS Arabic versions—MHLS-Arabic-Adolescents, MHLS-Arabic-General Public, and MHLS-Arabic-Students—as suitable instruments for assessing MHL among Arab-speaking populations globally. Our findings potentially contribute to the existing body of knowledge on MHL measurement by highlighting its inherent complexity and suggesting the importance of adopting micro-context-specific approaches for assessing MHL. This underscores the imperative to refine the MHLS to capture MHL’s nuances across diverse populations. Moreover, this study makes a notable contribution to the literature by providing three reliable modified versions of the MHLS in Arabic. This is particularly significant as it enables the study of MHL among the 436 million Arabic-speaking individuals worldwide who suffer from a high prevalence of mental health problems and disorders. Understanding MHL across different Arab demographic groups can facilitate the development of tailored intervention initiatives that aim to promote mental health service use, enhance mental health outcomes, and ensure equitable access to high-quality treatments. Finally, this research has an indirect yet equally important implication: the need for foundational exploration of MHL among Arabs. Understanding how MHL manifests within these communities is essential for developing new questionnaires, not just ones modified from existing ones but specifically tailored to assess MHL across diverse Arab groups.
